# Infrared Signatures of Phycobilins within the Phycocyanin 645 Complex

**DOI:** 10.1021/acs.jpcb.3c01352

**Published:** 2023-05-16

**Authors:** Partha Pratim Roy, Cristina Leonardo, Kaydren Orcutt, Catrina Oberg, Gregory D. Scholes, Graham R. Fleming

**Affiliations:** † Department of Chemistry, University of California, Berkeley, California 94720, United States; ‡ Molecular Biophysics and Integrated Bioimaging Division, Lawrence Berkeley National Laboratory, Berkeley, California 94720, United States; § Department of Chemistry, Princeton University, Washington Road, Princeton, New Jersey 08540, United States; ∥ Kavli Energy Nanoscience Institute at Berkeley, Berkeley, California 94720, United States

## Abstract

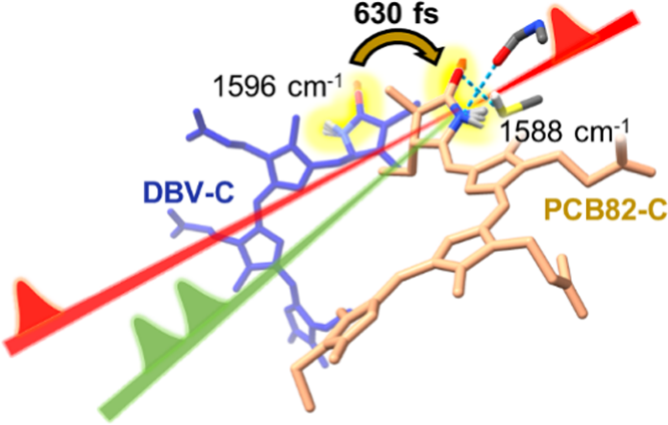

Aquatic photosynthetic organisms evolved to use a variety of light frequencies to perform photosynthesis. Phycobiliprotein phycocyanin 645 (PC645) is a light-harvesting complex in cryptophyte algae able to transfer the absorbed green solar light to other antennas with over 99% efficiency. The infrared signatures of the phycobilin pigments embedded in PC645 are difficult to access and could provide useful information to understand the mechanism behind the high efficiency of energy transfer in PC645. We use visible-pump IR-probe and two-dimensional electronic vibrational spectroscopy to study the dynamical evolution and assign the fingerprint mid-infrared signatures to each pigment in PC645. Here, we report the pigment-specific vibrational markers that enable us to track the spatial flow of excitation energy between the phycobilin pigment pairs. We speculate that two high-frequency modes (1588 and 1596 cm^–1^) are involved in the vibronic coupling leading to fast (<ps) and direct energy transfer from the highest to lowest exciton, bypassing the intermediate excitons.

## Introduction

Photosynthesis is the process by which solar energy is converted into fuel in photoautotrophic organisms.^1^ Photosynthetic organisms have evolved to use wavelengths of light that typically exist in their environment.^2−5^ Cryptophytes are widely studied algae, present in both freshwater and marine habitats. In aquatic environments, the red light of the solar spectrum is absorbed by water or chlorophyll-containing algae, and the blue light is scattered. Red light is often scarce, so the cryptophytes (like cyanobacteria and red algae) have evolved to utilize open-chain phycobilin chromophores absorbing the green part of the solar spectrum, in contrast to the light-harvesting complexes of the abundant green algae and higher plants that use closed-chain chlorophylls to absorb red light. Furthermore, depending on the environmental conditions, cryptophytes tune their absorption wavelengths by subtle modifications in chemical structures and protein linkage of the constituent phycobilin chromophores.^4,6,7^

**Figure 1:**
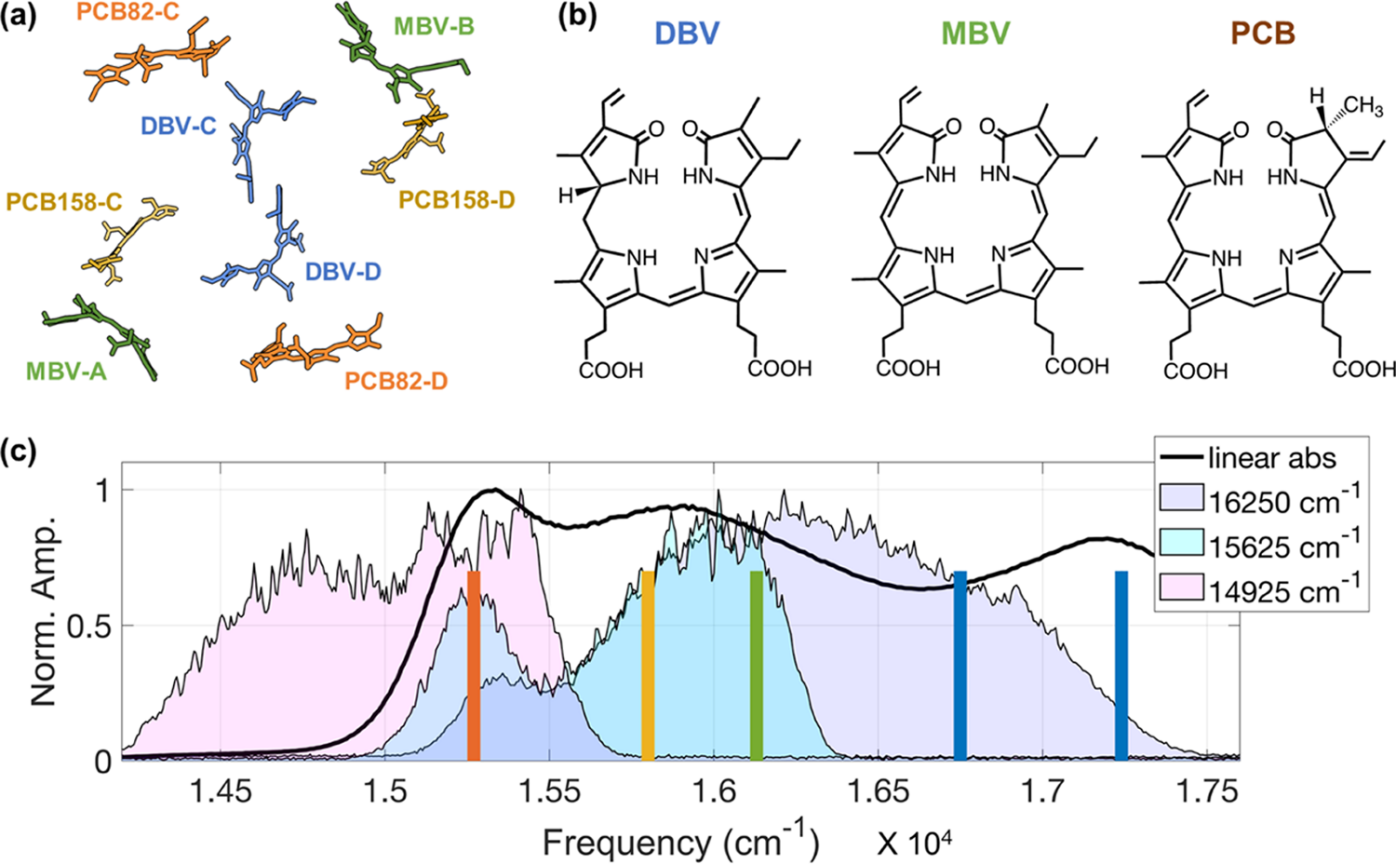
(a) Crystal structure of PC645 (4lms) shows the spatial arrangement of the biliverdin pigments.^33^ (b) Chemical structures of the three main biliverdin pigments: dihydrobiliverdin (DBV), mesobiliverdin (MBV), and phycocyanobilins (PCB). (c) Linear absorption spectrum of PC645 at 120 K is represented in a black solid line. Three different excitation spectra (centered at 16 250, 15 625, and 14 925 cm^–1^, respectively) are used for excitation-frequency-dependent pump-probe experiments, shown with different filled curves. The excitonic peak maxima are indicated by colored vertical lines according to literature values:^34^ PCB82 (orange), PCB158 (yellow), MBV (green), DBV–, and DBV+ (blue).

This work focuses on the phycobiliprotein phycocyanin 645 (PC645), a light-harvesting complex in the cryptophyte algae *Chroomonas mesostigmatica* (CCMP269).^6^ PC645 employs chromophores with diverse chemical structures as well as strong interpigment coupling to expand its spectral cross section for light absorption. Three structurally different phycobilin chromophores are found in PC645 (Figure 1a): two dihydrobiliverdin (DBV-C and DBV-D), two mesobiliverdin (MBV-A and MBV-B), and four phycocyanobilins (PCB158-C, PCB158-D, PCB82-C, and PCB82-D). PC645 down-converts the absorbed solar light (645 nm) and transfers to chlorophyll-based antennas (677 nm)^8^ with over 99% efficiency.^9^ The sub-ps interpigment energy transfer aids this efficient light harvesting. Most interestingly, the direct energy transfer from DBVs to PCB82, spanning an energy gap over 1500 cm^–1^, happens within 0.6–0.8 ps.^10^ Such a fast energy transfer is explained by the interplay of interpigment electronic coupling and spectral overlap (i.e., they are not separable on the timescales relevant to the energy transfer), suggesting the involvement of vibronic coupling.^11,12^

Vibrations play a crucial role in excitation energy transfer, like that from the DBVs to PCB82, because the vibronic progressions in the donor emission spectrum and/or the acceptor absorption spectrum dominate the spectral overlap integral. Without these (Raman-active) vibrations, energy conservation during energy transfer would be ensured only by overlap of the tails of those spectra, and therefore it would be quite slow. Vibrations of the chromophores and surrounding protein also play a role in energy transfer by contributing to the reorganization energy. One way of thinking about these modes is that they provide “friction” that limits the rate of energy transfer. They also play an important role in localizing excitation.

Two-dimensional electronic spectroscopy study by Scholes and co-workers has shown evidence of a fast (∼100 to 200 fs) coherence-mediated energy transfer within the strongly coupled DBV exciton pair.^13,14^ Coherent oscillations of the frequency bridging the DBV-PCB energy gap were observed to survive more than 400 fs at ambient temperature and were assigned, by comparison with calculations, to indicate a vibronic coherence. Multiple theoretical studies have predicted that the vibrational motion of the chromophores involved is correlated by electronic coupling, which leads to such long-lived quantum coherence.^13,15−18^ The energy transfer between PCB pigments, on the other hand, are rather slow processes, ranging from ∼5 ps (PCB158 → PCB82) to ∼45 ps (PCB82-D → C), which falls in the incoherent Förster regime.^10^ Overall, it suggests that the vibrational dynamics arising from the local protein environment determine the pathways of excitation energy transfer in PC645. The present work focuses on these various vibrations and their interplay with energy transfer in PC645.

The IR vibrational spectra give insight into the local protein environment. Although the vibrational spectra of the isolated phycobilin pigments can be readily measured,^19^ strong coupling between the chromophores can significantly modify the vibrational frequencies from their uncoupled values.^20,21^ In addition, conformational changes induced by the protein, as well as electrostatic, hydrogen bond, and steric interactions, can affect the vibrational modes of each pigment. Moreover, the overwhelming amide(I) and amide(II) absorption bands from the protein obscure the mid-IR region^22,23^ relevant to the largest energy gap in PC645 in the absence of a selective perturbation, such as optical excitation. In this work, we take advantage of the selectivity of two-dimensional electronic vibrational (2DEV) spectroscopy combined with visible-pump-IR-probe spectroscopy to characterize key vibrational frequencies reporting on energy transfer in intact PC645. In both techniques, we excite the system with visible pulses and probe with an IR pulse to identify the mid-infrared signatures of each phycobilin pigment within PC645 at 120 K. Visible-pump IR-probe is used to track the dynamics from different chromophores. Despite the wide energy gaps between each excitonic state, their bandwidths lead to spectral overlap which makes the assignment of infrared signatures to specific pigments difficult via pump-probe data. We, therefore, take advantage of the excitation-energy-dependent infrared signatures of 2DEV spectroscopy, which, combined with the enhanced spectral resolution of this technique,^24−30^ allows us to identify the infrared features specific to each pigment in the PC645 complex. 2DEV spectroscopy ultimately provides a detailed picture of the spatiotemporal flow of the excitation energy between each pigment pair in PC645 due to the influence of the local protein environment on the IR signatures of each pigment.

## Experimental Methods

### PC645 Sample Preparation and Composition

PC645 phycobiliproteins were isolated from *C. mesostigmatica* (CCMP269) cryptophyte algae that were cultured in Prov50 media (NMCA) at room temperature under a 12-h light/12-h dark cycle. Algae were harvested by centrifugation at 2000*g* at 10 °C for 2 min and resuspended in 100 mM phosphate buffer (pH 7.2). The resuspended algae were frozen at −20 °C for at least one day before purification of the protein using gradual ammonium sulfate precipitation. The precipitated proteins were collected using ultracentrifugation at 35 000 rpm at 4 °C for 30 min (Beckman Coulter, Ti-60 rotor), producing a protein pellet. The protein pellet was resuspended in 100 mM deuterated phosphate buffer followed by a buffer exchange to produce concentrated PC645 in 25 mM deuterated phosphate buffer.

The sample was stored at −80 °C after purification, then thawed and concentrated prior to spectroscopic analysis. The sample concentration has been performed using Amicon Ultra-0.5 Centrifugal Filter Unit (MilliporeSigma). The sample was centrifuged at 11 000 rpm for 14 min and then reverse spun at 1000 rpm for 6 minutes to collect the concentrated sample. Approximately 500 μL of sample has been concentrated to about 25 μL with 20 times concentration. The concentrated PC645 sample was then diluted in a 1:2 (v/v) ratio with deuterated glycerol for better glass formation at 120 K. After dilution with glycerol, the sample was stored at −4 °C and used for the experiment within 2 weeks.

### Visible-Pump IR-Probe and 2DEV Experiments

A Ti/sapphire-based setup has been used for the visible-pump IR-probe and 2DEV measurements. The setup is described elsewhere.^26,29^ Briefly, a Ti/sapphire oscillator (Vitara-S, Coherent) was regeneratively amplified (Legend, Coherent) with a 1 kHz repetition rate. The amplified pulse was divided into two beams: one beam was used to pump a home-built noncollinear optical parametric amplifier (NOPA); the other beam was used to generate a mid-IR-probe pulse (1580–1780 cm^–1^) with an optical parametric amplifier-difference frequency generation home-built setup. The NOPA output was compressed to ∼16 fs with a prism pair and a pulse shaper (Dazzler, Fastlite). The Dazzler was used to tune the excitation pulse central frequency and bandwidth. The visible pulse was focused on the sample to a spot size of ∼250 μm in diameter. The cross-correlation between the visible and mid-IR pulses showed a ∼100 fs instrument response time. The mid-IR pulse was split by a 50:50 beam splitter to produce a probe and a reference beam. Both IR beams were focused on the sample to a spot size of ∼200 μm in diameter. After passing through the sample, the mid-IR beams were dispersed with a spectrometer (Triax 180, Horiba) onto a dual-array 64-pixel HgCdTe detector (Infrared Systems Development). The delay between the visible and IR pulses was controlled by a motorized translation stage. The delay was scanned from −500 fs to 2 ps with 20 fs time steps, from 3 to 50 ps with 1 ps time steps, and from 55 to 100 ps with 5 ps time steps. Global analysis was performed on the pump-probe data to determine the evolution-associated difference spectra (EADS) and the corresponding time constants using Glotaran.^31^

2DEV spectroscopy has been performed with the same experimental setup, using the Dazzler to generate a pair of visible pulses with relative delay time scanned between 0 and 100 fs with 2.5 fs time steps. Fourier transform has been applied to reconstruct the excitation axis. The visible pulses had a central wavelength of 16 250 cm^–1^ (15 100–17 500 cm^–1^) and a combined energy of ∼80 nJ. Because of the pump-probe geometry, a 3 × 1 × 1 phase cycling scheme has been applied to reconstruct the rephasing and non-rephasing signals.^32,33^ All pump-probe and 2DEV measurements have been performed at cryogenic temperature (120 K) using an optical cryostat (OptistatDN2, Oxford Instruments). The optical density of the sample at 650 nm was 0.65 with an optical path length of 100 μm. To avoid photobleaching, the sample spot was changed between each set of measurement.

## Results and Discussion

### Excitation-Frequency-Dependent Transient IR Spectroscopy

PC645 is an (αβ)_2_ homodimer with a closed-form quaternary structure.^34^ The pigments in PC645 are open-chain tetrapyrroles (bilin chromophores) covalently bound to cysteine residues in the phycobiliprotein. Within each monomer, the α-subunit binds a mesobiliverdin (MBV), while the β-subunits bind a dihydrobiliverdin (DBV) and two phycocyanobilins (PCB82 and PCB158). The spatial arrangement of the pigments inside the protein is depicted in Figure 1a. Figure 1b shows the chemical structures of PCB, MBV, and DBV pigments. The mid-IR spectrum ranging from 1580 to 1780 cm^–1^ is used to probe the C=N, C=C, and C=O stretching vibrations, which are well-known sensitive reporters of local protein environment.

**Figure 2:**
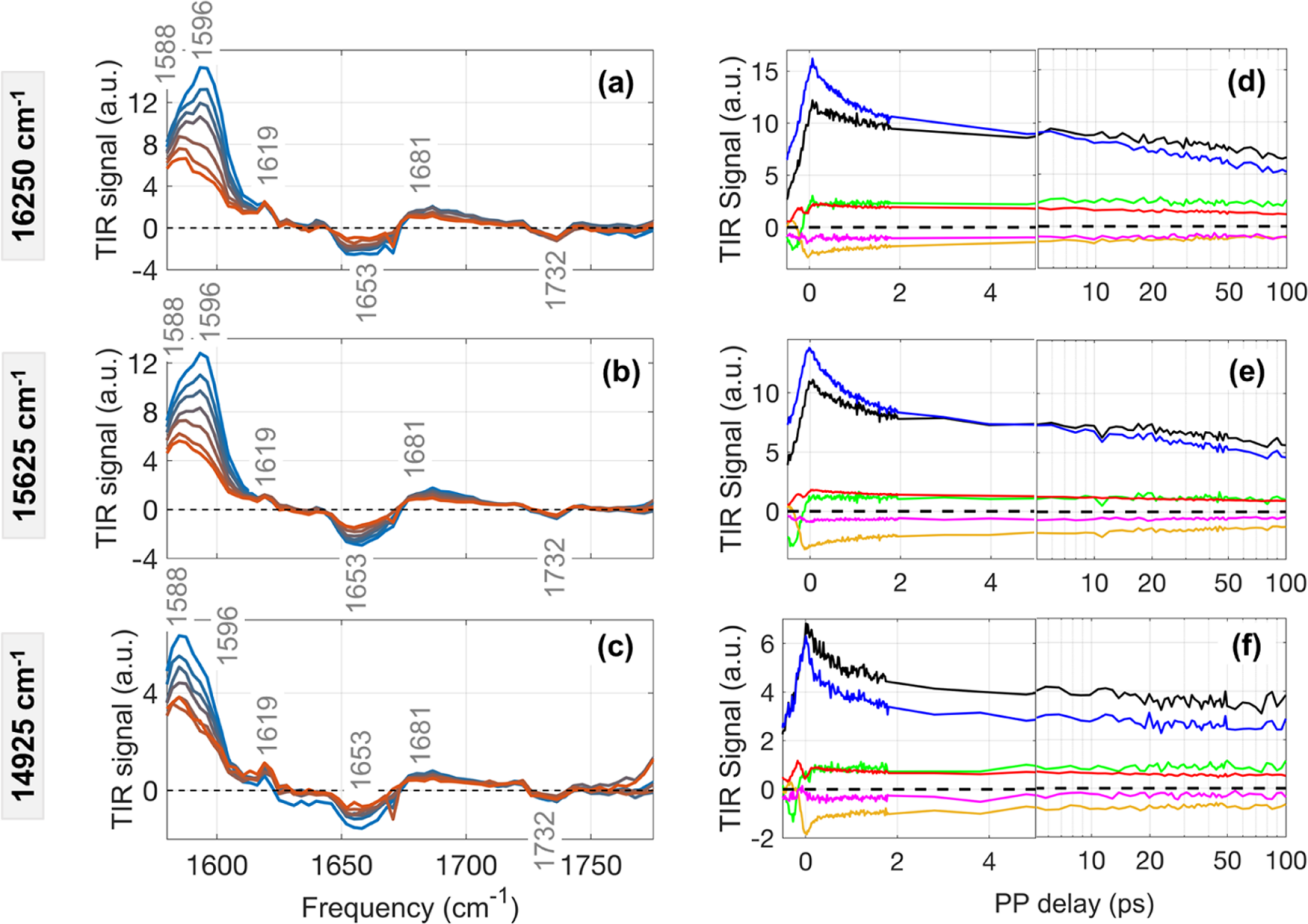
(a–c) Transient IR spectra with increase (blue to orange) in pump-probe delays (0.2, 0.5, 1, 2, 5, 10, and 50 ps) observed with the three different excitation spectra centered at 16 250, 15 625, and 14 925 cm^–1^ shown in Figure 1c. (d–f) Time evolutions of the IR bands at 1588, 1596, 1619, 1653, 1681, and 1732 cm^–1^ are shown in black, blue, green, yellow, red, and magenta, respectively.

The electronic site energies of the constituent pigments are well separated due to their different electronic structures. The DBV dimer is strongly coupled (320 cm^–1^), forming the delocalized exciton pair DBV+ (upper) and DBV– (lower) with a large inter-exciton energy gap (620 cm^–1^).^13,14,35^ The DBV pigments are weakly coupled with the MBV (42 cm^–1^) and PCBs (25–30 cm^–1^). The electronic coupling between MBV and the PCBs is stronger (PCB158 ∼ 85 cm^–1^; PCB82 ∼ 55 cm^–1^) while that between PCB158 and PCB82 is much weaker (11 cm^–1^).^13,14^Figure 1c illustrates the linear absorption spectrum of PC645 at 120 K and the underlying five excitonic levels (DBV+, DBV–, MBV, PCB158, and PCB82), as reported in the literature.^11,35^ The combination of coupling and site energies lead to excitonic levels that are well separated. The largest energy gap is between the DBV and PCB excitonic levels, at approximately 1500 cm^–1^. We take advantage of this energy separation to analyze the dynamics from specific groups of pigments by selectively exciting the corresponding excitonic levels. The visible-pump IR-probe measurements were performed with three different excitation spectra (Figure 1c) with center frequencies of 16 250 cm^–1^ (spectral range 15 100–17 500 cm^–1^), 15 625 cm^–1^ (15 000–16 400 cm^–1^), and 14 925 cm^–1^ (14 400–16 000 cm^–1^). The excitation-frequency-dependent transient IR spectra are illustrated in Figure 2. The 16 250 cm^–1^ excitation spectrum covers all five major excitonic manifolds (DBV+, DBV–, MBV, PCB158, and PCB82), and thus the evolution of corresponding pump-probe spectra is the convolution of all possible excitonic relaxation pathways (Figure 2a,d). The excitation spectrum centered at 15 625 cm^–1^ covers only the MBV and PCB levels, removing the pathways arising from the direct excitation of the DBV pigments (Figure 2b,e). The spectrum centered at 14 925 cm^–1^ covers only the PCB levels, removing the pathways from the MBV and DBV pigments (Figure 2c,f).

Ground state bleach (GSB) and excited state absorption (ESA) in the transient IR spectra appear as positive and negative peaks, respectively (Figure 2a–c). The GSB peaks appearing at 1588, 1596, 1619, and 1681 cm^–1^ are in close agreement with the FTIR spectrum reported for isolated PCB in solution.^19^ The IR peaks above and below 1600 cm^–1^ are assigned as C=O and C=NH^+^ stretch in the literature.^19,38,39^ In Figure 2d–f, the offset in each transient represents the remaining population in the lowest exciton (PCB82), which returns to the ground state on a nanosecond timescale.^36,37^ The IR bands of DBV/MBV and the PCBs can be qualitatively distinguished based on their dynamics. Those bands that decay on sub-ps timescales can be assigned to DBV or MBV pigments, while the signatures that do not decay within our experimental timescale are assigned to the lowest excitonic state, PCB82.

Analysis of the temporal evolution of the two strongest GSB peaks at 1588 and 1596 cm^–1^, previously attributed to C=NH^+^ stretching modes,^11,38,39^ reveals excitation-frequency-dependent dynamics. In the transient IR spectra with the excitation spectrum centered at 16 250 and 15 625 cm^–1^ (Figure 2d,e), the 1596 cm^–1^ peak is stronger at initial times (<2 ps) but decays more rapidly than the 1588 cm^–1^ peak, which becomes the strongest peak after 2 ps. In contrast, the transient IR spectra with the excitation spectrum centered at 14 925 cm^–1^ (covering only the PCBs) show that the 1588 cm^–1^ band is stronger than the 1596 cm^–1^ band at short times (<ps) and that both bands decay on the same timescale (Figure 2f). Based on these dynamics, the peak at 1596 cm^–1^ is assigned to all three bilins (DBV/MBV/PCB) but appears stronger for the DBV/MBV pigments, while the peak at 1588 cm^–1^ is specific to the PCB82 pigments. The sub-ps dynamics of the 1588 cm^–1^ PCB82 mode indicate vibronic mixing character with the higher energy bilins and will be discussed in more detail in the last section. All of the other bands above 1600 cm^–1^ are attributed to C=N, C=C, and C=O stretching modes.^19,38,39^ Among them, the GSB band at 1619 cm^–1^ and ESA band at 1732 cm^–1^ do not decay in 100 ps and, therefore, can be assigned to PCB82. On the other hand, the GSB band at 1681 cm^–1^ and the ESA band at 1653 cm^–1^ have a sub-ps decay and may also be signatures of DBV and/or MBV.

**Figure 3:**
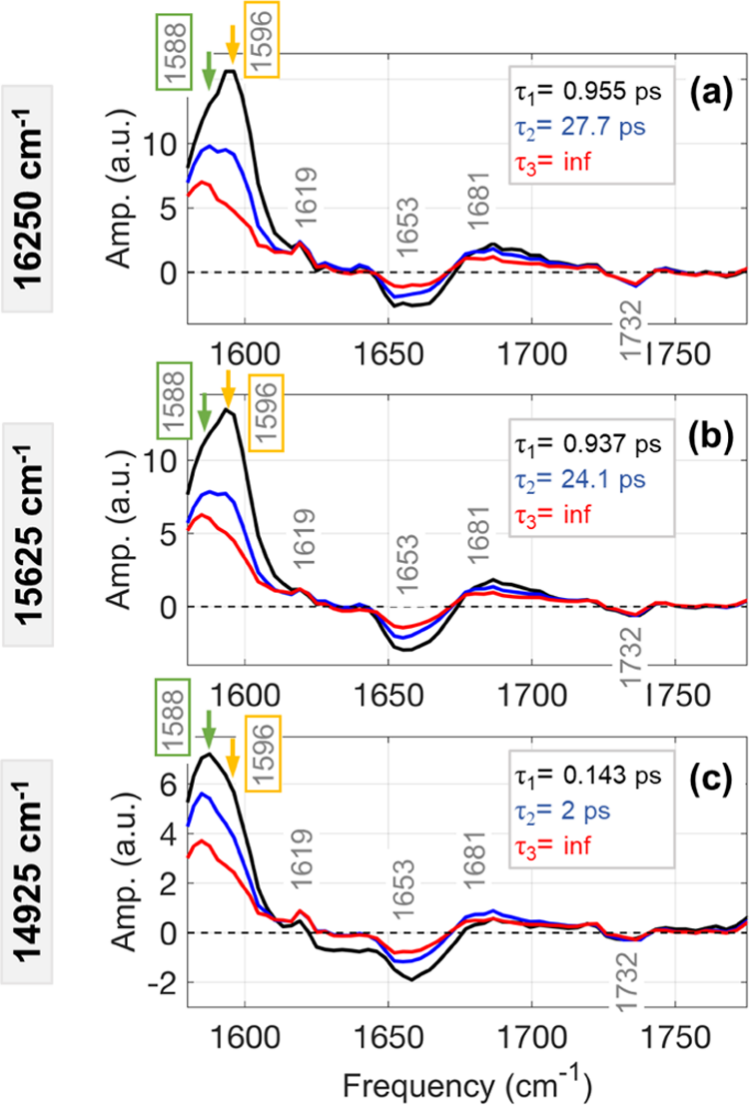
Evolution-associated difference spectra obtained from the global analysis of the transient IR spectra obtained with three different excitation spectra shown in Figure 1c. The GSB peaks at 1588 and 1596 cm^–1^ peaks are marked with green and yellow arrows, respectively, to highlight the change in their relative intensities.

In addition to the qualitative assignment of IR signatures from the excitation-frequency-dependent pump-probe measurements, the temporal evolution of these signatures can be analyzed. The dynamical information is obtained by performing global analysis on the pump-probe spectra using Glotaran.^31,40^ Each excitation is fit with three exponential components using the sequential model, which gives the evolution-associated difference spectra (EADS, Figure 3). The third time constant is fixed to a value much longer than the experimental time (∼100 ps) to account for the dynamics beyond the scale of the experiment. The pump-probe data recorded with excitation at 16 250 cm^–1^ and at 15625 cm^–1^ give similar time constants of 950 fs and 26 ps (Figure 3a,b). The 950 fs component corresponds to the direct energy transfer from the DBV and/or MBV to the lowest excitonic level, PCB82, both reported to occur on a sub-picosecond timescale.^10,35,41^ The 26 ps time constant in our data is most likely the convolution of two sequential pathways (PCB158 → PCB82 and PCB82D → PCB82C). Marin et al.^10^ reported that the energy transfer from PCB158 to PCB82 happens in 5–7 ps and is followed by an interunit energy hopping from PCB82D to PCB82C, which occurs in approximately 45 ps. Since the DBV and MBV are almost equally coupled to the PCBs, the nearly identical dynamics obtained for both excitations (16 250 and 15 625 cm^–1^) are reasonable.

The pump-probe signal recorded with the red-most excitation at 14 925 cm^–1^ gives time constants of 140 fs and 2 ps (Figure 3c). The 140 fs component is very close to our instrument response time (∼100 fs) and it is not considered for the interpretation of the dynamics within PC645. The 2 ps component is assigned to the transition from PCB158 to PCB82, which is reported to occur in approximately 5 ps.^10^ Unlike the two blue excitations, the slow energy transfer from PCB82-D to PCB82-C (∼45 ps) is not observed, most likely because of the reduced signal-to-noise ratio in our data at the longer pump-probe delay (>10 ps), which limits the ability of the global analysis program to fit slower dynamics.

The excitation-frequency-dependent pump-probe measurements allow us to qualitatively assign the IR signatures to specific subgroups of pigments. The EADS obtained from global analysis provides information on the evolution of each IR signature. However, the spectral broadening produces significant spectral overlap even though the electronic excitons are well separated in energy. This limits our ability to identify the specific IR signatures of each pigment quantitively. We address this issue by combining the dynamical information obtained from the pump-probe data with the 2DEV spectroscopy study discussed next.

### 2DEV Spectroscopy

**Figure 4:**
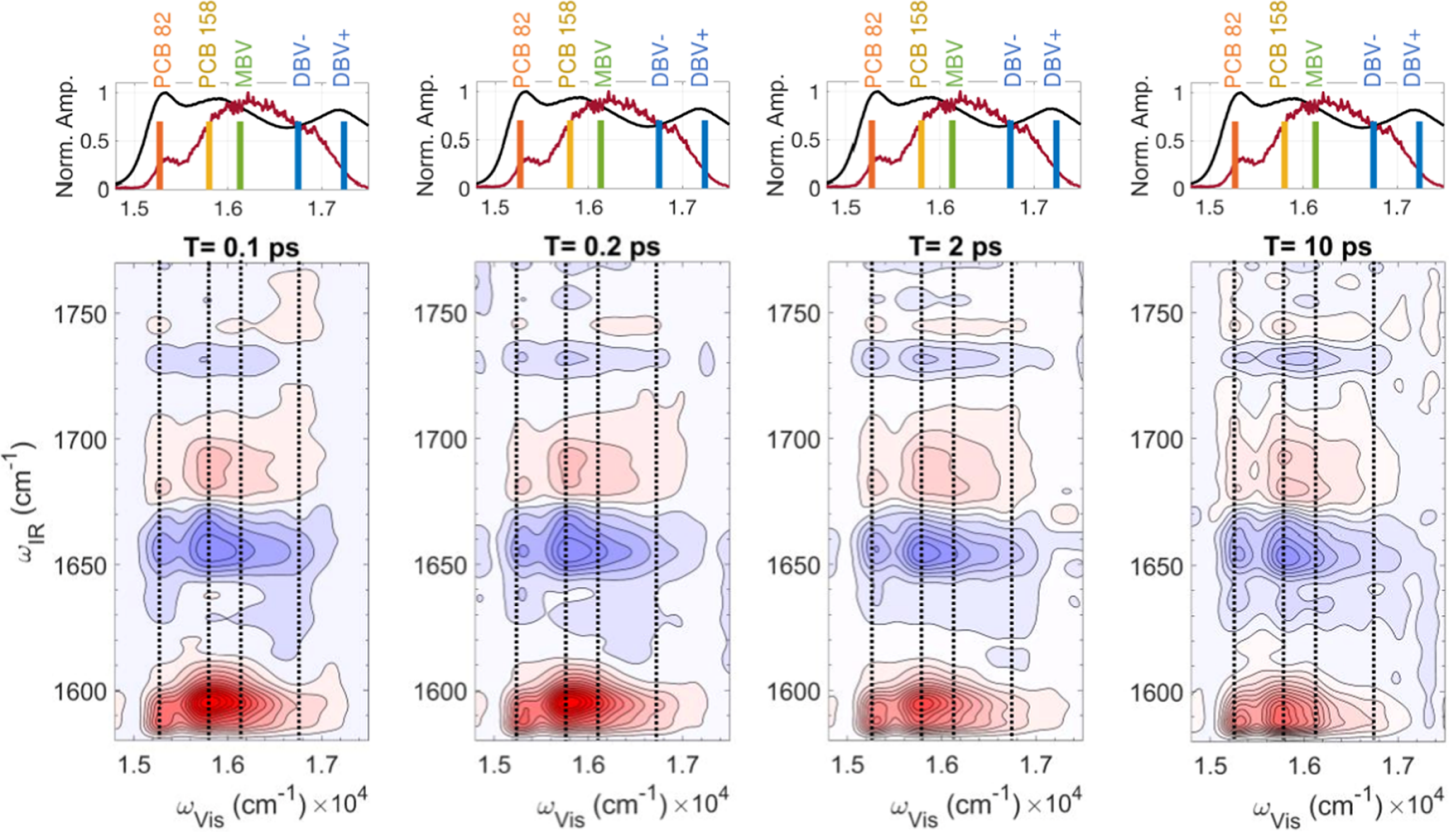
2DEV spectra of PC645 at 120 K at four selected waiting times *T* = 100 fs, 200 fs, 2 ps, and 10 ps. The dotted vertical lines represent the excitonic peak maxima. The top panel of each 2D graph shows the normalized linear absorption spectrum (black) of PC645 at 120 K and the broadband excitation spectrum (brown) covering all of the excitons shown with colored vertical lines. The excitonic peak maxima are indicated by the colored solid vertical lines: PCB82 (orange), PCB158 (yellow), MBV (green), DBV–, and DBV+ (blue).

**Figure 5:**
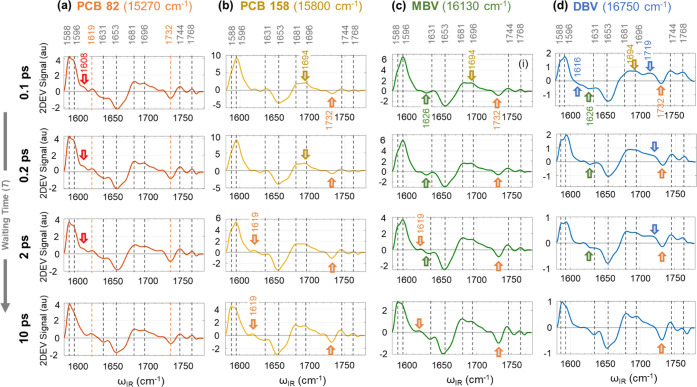
2DEV spectra at the excitation frequencies corresponding to the exciton peak maxima (a) DBV (16 750 cm^–1^), (b) MBV (16 130 cm^–1^), (c) PCB158 (15 800 cm^–1^), and (d) PCB82 (15 270 cm^–1^) at four waiting times (top to bottom: 100 fs, 200 fs, 2 ps, 10 ps). The vertical gray dotted lines represent the IR bands that commonly appear in all excitons. The orange dotted lines in (a) represents the marker band for PCB82. The IR bands that show major change in spectral features with an increase in waiting time are highlighted with colored arrows. The blue, green, yellow, orange, and red colors indicate the characteristic IR bands for DBV, MBV, PCB158, PCB82-D, and PCB82-C, respectively.

2DEV spectroscopy, which provides enhanced spectral resolution by adding information along the excitation frequency axis, is used to distinguish the IR signatures of each pigment.^28,29^ The excitation spectrum used for 2DEV measurements is the same broadband spectrum used for pump-probe experiment centered at 16 250 cm^–1^ covering all five excitons (Figure 1c). Taking advantage of the dynamical information obtained from pump-probe spectra, we selected four waiting times for the 2DEV measurements: 100 fs, 200 fs, 2 ps, and 10 ps (Figure 4). In order to avoid photodamage of the sample and achieve a high signal-to-noise ratio (see Figure S3), we only focused on a few waiting times by averaging 16 000 laser shots to collect a 2DEV spectrum for each selected waiting time. The IR peaks indicated in red and blue represent GSB and ESA bands, respectively. The four dotted lines represent the maxima of four major excitonic levels (DBV–, MBV, PCB158, and PCB82) covered by our broadband excitation spectrum (Figure 1c, red). In general, most of the spectral signatures across the excitation frequency axis appear to be similar, which is expected considering the similarity of the chemical structures of the three chromophores (Figure 1b). However, closer inspection of the spectra shows that there are a number of IR peaks (highlighted with arrows in Figure 5), which differ significantly at early waiting times (e.g., 0.1 ps) and become more similar within 10 ps. For example, at *T* = 0.1 ps, the relative intensity of the 1596 cm^–1^ band is greater than that of the 1588 cm^–1^ peak at the MBV excitonic maximum while it is weaker at the PCB82 excitonic maximum. At a longer waiting time, *T* = 10 ps, the relative intensity of the GSB band at 1596 cm^–1^ is lower than that of the GSB band at 1588 cm^–1^ for both the excitonic maxima of MBV and PCB82. This indicates that the population transfers to the same final state (PCB82) regardless of the initial state after excitation.

For a selected waiting time, the 2DEV spectral slice at the excitation frequency (ω_vis_) at each excitonic peak maximum gives insight into the IR structure of the corresponding pigment/exciton (Figure 5). We use our knowledge about the inter-excitonic relaxation dynamics obtained from the visible-pump IR-probe study to characterize each IR band. Below we discuss the evolution of 2DEV spectra at different excitation frequencies starting from lowest (PCB82) to highest (DBV) energy excitonic peak maxima. Note that some of the features in Figure 5 are quite small, but exceed the noise floor in our measurement. Details of the determination of the noise level are given in the Supporting Information, which expressed in the same unit as Figure 5 is <0.1.

#### PCB82

The 2DEV spectral slice at the excitonic maximum of PCB82 (ω_vis_ = 15 270 cm^–1^) does not show spectral evolution with increasing waiting time (Figure 5a), as expected for the lowest energy excitonic state. Like the transient IR spectra (Figure 2), the GSB band at 1619 cm^–1^ and an ESA at 1732 cm^–1^ have nearly same amplitude at all waiting times. Therefore, we assign these two IR peaks (highlighted in orange) as marker bands for PCB82. A minor change is observed at 1608 cm^–1^, which appears as a shoulder at the initial time and is prominent up to 2 ps. At 10 ps, it is overshadowed by the adjacent strong 1596 cm^–1^ band, showing a slower decay. Similarly, at 10 ps the ratio between the peaks at 1588 and 1596 cm^–1^ changes, showing the rise of the 1588 cm^–1^ peak. As the final inter-monomer energy hopping from PCB82-D to PCB82-C takes place in ∼45 ps,^10^ we attribute this IR feature at 1608 and 1588 cm^–1^ to PCB82-D and C, respectively.

#### PCB158

We can compare the characteristic IR signatures of PCB158 and PCB82 by comparing 2DEV spectral slices at the excitonic peak maxima of PCB158 (ω_vis_ = 15 800 cm^–1^, Figure 5a) and PCB82 (ω_vis_ = 15 270 cm^–1^, Figure 5b) at early waiting times (e.g., 0.1 ps). Most of the IR peaks appear identical in PCB158 and PCB82 as they both have the same chemical structure. Two major differences appear in the relative intensity of (i) the GSB peak pair at 1588 vs 1596 cm^–1^ and (ii) the GSB peak pair at 1681 vs 1696 (PCB82)/1694 (PCB158) cm^–1^. For PCB82 (Figure 5a), the 1588 and 1681 cm^–1^ peaks appear stronger compared to the 1596 and 1696 cm^–1^ peaks, respectively, while the opposite is seen for PCB158 (Figure 5b). With an increase in waiting time, the relative intensities of those pairs of IR bands (1588/1596 and 1681/1694 cm^–1^) are reversed. In addition, the 1694 cm^–1^ peak gradually shifts to 1696 cm^–1^ at longer waiting times. Furthermore, the peaks at 1619 and 1732 cm^–1^ that are assigned to PCB82 grow in, indicating energy transfer from PCB158 to PCB82. At 10 ps, the IR signature matches the signature found at early waiting times at the PCB82 peak maximum, indicating energy transfer from PCB158 to PCB82 is complete, in agreement with the 5 ps time constant reported in the literature.^10,35^ The dynamics suggest that the peak at 1694 cm^–1^ belongs to PCB158. However, we note that the 1696 cm^–1^ peak assigned to PCB82 is very close to the 1694 cm^–1^ peak, making the attribution to a specific pigment somewhat ambiguous.

#### MBV

At the excitonic peak maximum of MBV (ω_vis_ = 16 130 cm^–1^, Figure 5c), an ESA peak at 1626 cm^–1^ appears at early waiting time; this peak is not observed at the PCB82 maximum and appeared weakly for PCB158 maximum, which may arise from spectral overlap with the close-lying MBV, and therefore, we attribute 1626 cm^–1^ peak predominantly to the MBV pigments. The 1626 cm^–1^ peak shifts to 1631 cm^–1^ resembling PCB82 at later waiting times, indicating the MBV relaxation. Like PCB158, the MBV relaxes to PCB82 on sub-ps (∼0.8 ps)^10^ timescale, so all of the characteristic bands and their spectral evolution observed exciting PCB158 (highlighted in yellow) are also present at the MBV peak maximum. Within 10 ps the exciton relaxes to PCB82 and hence shows the same IR signatures observed for the PCB82 exciton.

#### DBV

At the DBV excitonic maximum (ω_vis_ = 16 750 cm^–1^, Figure 5d), a new ESA peak at 1616 cm^–1^ and a GSB peak at 1719 cm^–1^ are observed that are not seen for other excitons, and therefore are attributed to the DBVs. In addition, the presence of a PCB82 marker ESA peak at 1732 cm^–1^ is observed already at early waiting time showing evidence of an ultrafast and direct energy transfer from DBV to PCB82 in spite of the large excitonic energy gap. At 10 ps, like other excitons, the 2DEV spectral slice shows features of PCB82.

### Assignment of IR Peaks

Using the dynamical information of pump-probe and 2DEV spectroscopy data of PC645, we identify the infrared signatures specific for each pigment in their native protein environment. The peaks at 1616 cm^–1^ (ESA) and 1719 cm^–1^ (GSB) are specific to the DBV pigments, while the peak at 1626 cm^–1^ (ESA) is specific to MBVs and the one at 1694 cm^–1^ (GSB) is specific to the PCB158. Furthermore, the bands at 1619 (GSB) and 1732 cm^–1^ (ESA) belong to both PCB82 pigments, while the peaks at 1588 and 1608 cm^–1^ (GSB) are specific to PCB82-C. The results of our assignments of the mid-IR GSB/ESA frequencies to specific bilins are given in Table 1. In particular, we focus on C=N, C=C, and C=O stretching modes. The subtle differences in the chemical structures of these pigments (Figure 1a) affect the corresponding vibrational frequencies, especially the highly localized modes (>1000 cm^–1^).^39^ In addition, different electrostatic interactions with the protein are expected to further shift the frequency of the identifying “fingerprint” vibrational modes. Here, we attempt to understand the influence of chemical structure and protein environment on the observed specific infrared signatures. In particular, we focus on the hydrogen bonds of the C=NH^+^ and C=O groups of each pigment with the protein side chains. These observations are based on the structural information found in the RCSB Protein Data Bank (PDB ID: 4lms)^34^ and on the vibrational modes attributions reported in the literature.^19,38,39^ The pigment–protein hydrogen bonds are used to address the specific vibrational frequencies seen for each pigment and are discussed in more detail in the Supporting Information. Hydrogen bonds with water are not considered here. The PCB and MBV pigments are reported to interact with water more than the DBV pigments, thus red-shifting the stretching modes more.^42^ Other pigment–protein electrostatic interactions are not considered here and might further impact the vibrational frequencies observed.

**Table 1:**
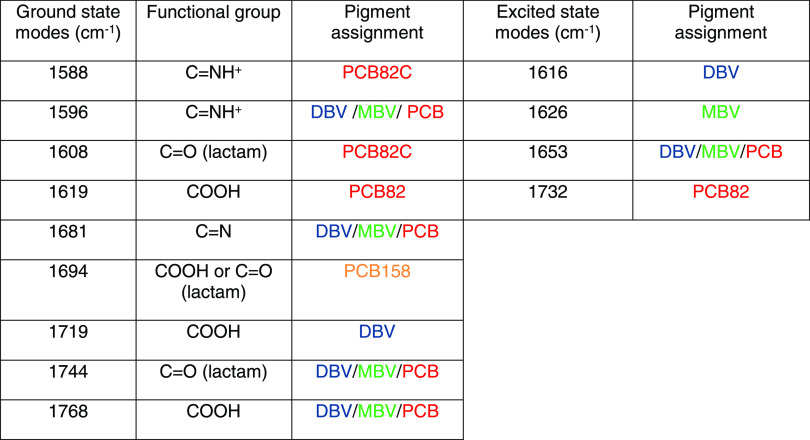
IR Peak Assignments Based on Transient IR and 2DEV Spectra

The two most external rings of the pigments are unsaturated γ-lactams (cyclic amides). Amide groups have two characteristic vibrational signatures in the IR region under investigation in this work: the amide(I) and the amide(II). The amide(I) (1650–1750 cm^–1^) is mainly the stretching vibrations of the C=O (70–85%) with some contributions from the C–N group (10–20%). The amide(II) (1580–1650 cm^–1^) is the combination of in-plane N–H bending (40–60%), C–N (18–40%), and C–C (10%) stretching vibrations. The γ-lactam amide(I) band (C=O stretching mode) is at slightly higher frequencies, between 1700 and 1750 cm^–1^. The peak observed for all pigments at 1744 cm^–1^ is attributed to this mode. The signature specific to PCB82-C at 1608 cm^–1^ is likely the C=O vibrational mode red-shifted by the hydrogen bond with a cysteine residue. The amide(II) band (mainly N–H stretching modes) is found at 1596 cm^–1^ for all pigments. The peak at 1588 cm^–1^ is the same mode, specific to PCB82. This peak is likely red-shifted by the hydrogen bond with *n*-methyl asparagine residue.

The C=N groups vibrate at 1640–1690 cm^–1^. Since those groups do not interact with the proteins strongly, we expect similar signatures for all pigments, likely the common signature at 1681 cm^–1^. The carboxylic groups have typical vibrations at 1760 cm^–1^, which corresponds to the 1768 cm^–1^ seen for all pigments. The signature at 1719 cm^–1^ characteristic of DBV is tentatively assigned to a red-shifted vibration of the carbonyl group forming a hydrogen bond with an arginine residue. This suggests that the band at 1719 cm^–1^ is specific to DBV-C. The band at 1619 cm^–1^ seen for PCB82-C and D is assigned to a carboxylic mode red-shifted by the hydrogen bonds with arginine residues for both pigments. The peak specific to PCB158 (1694 cm^–1^) can be attributed to a red-shifted peak of the carboxylic groups or of the C=O in the lactam rings, both forming hydrogen bonds with the protein side chains.

### Energy Transfer from DBV to PCB82

The 1732 cm^–1^ peak is assigned as a PCB82 exited state mode and is observed at sub-ps waiting times at the DBV excitonic maximum in 2DEV spectra (Figure 5d). This provides spectral evidence of direct energy transfer from the highest (DBV) to the lowest (PCB82) exciton, bypassing the other excitons on the energy ladder. These results are consistent with prior reports of efficient downhill energy transfer from DBV to PCB on sub-ps timescales.^10,11,35^

**Figure 6:**
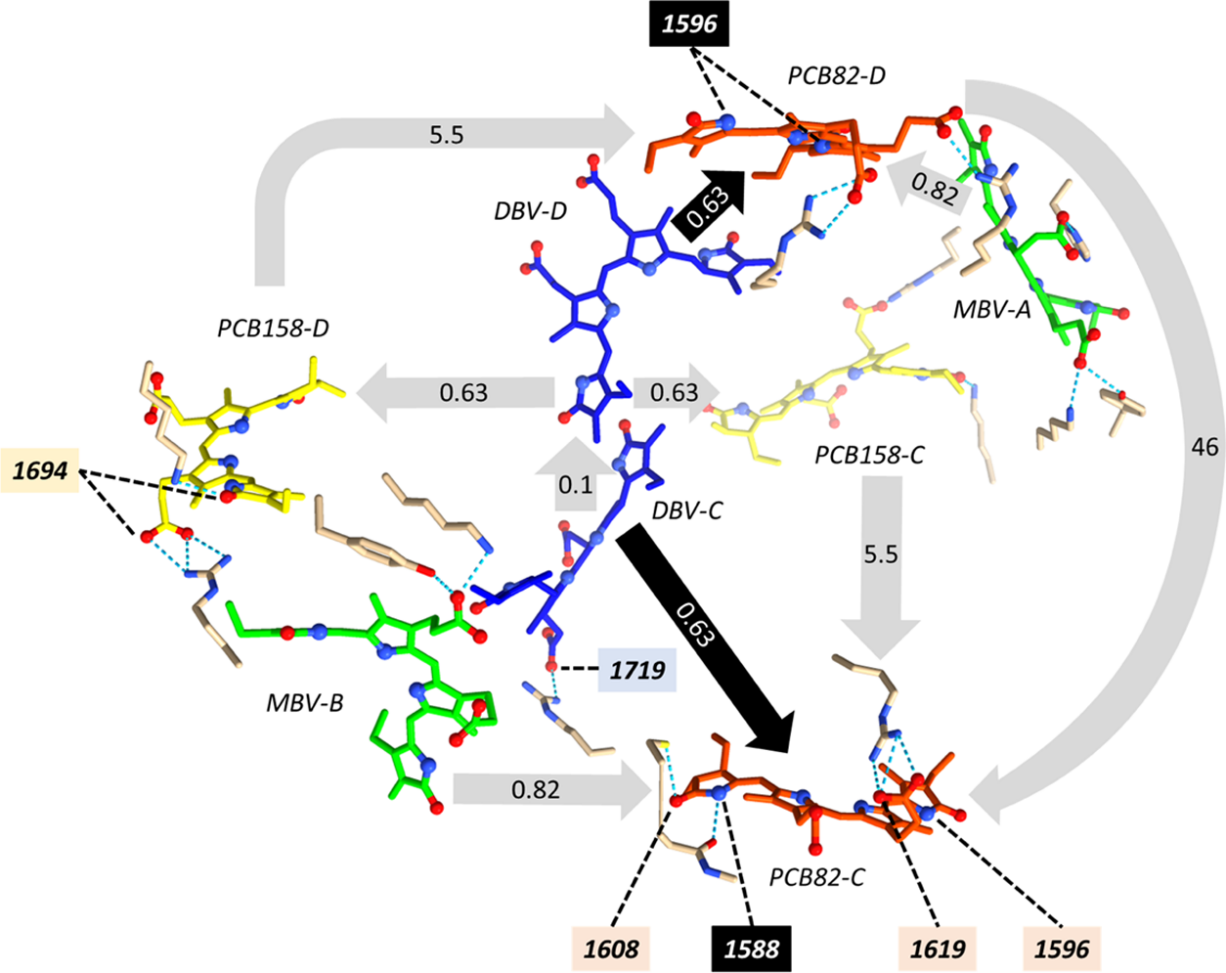
Schematic representation of the interpigment energy flow in PC645 (time constants within arrows, in picoseconds), as reported by Marin et al.^10^ with the marker infrared signatures specific to each pigment we reported in this study (bold italic number within boxes, in wavenumbers). Only hydrogen bonds with side chain amino acids are shown (PDB: 4lms).^33^

In addition, previous works reported the involvement of high-frequency vibrational modes, resonant to the energy gap between DBV and PCB82 (1580 cm^–1^), resulting in vibronic coupling and accelerating the rate of energy transfer.^11,12,43−45^ Our study finds a strong mode in PCB82 at 1588/1596 cm^–1^, assigned to C=N stretching strongly coupled with N–H out-of-plane bending,^39^ located very close to DBV (17 Å, Figure 6). This N–H out-of-plane bending vibration could modulate the interpigment distance and hence, the electronic coupling. In particular, our study shows PCB82 specific modes at 1588 and 1619 cm^–1^ while the 1596 cm^–1^ mode belongs to all pigments including DBV. This agrees with the broadband pump-probe study by Dean et al.,^11^ where they reported coherent Raman oscillation with frequencies of 1580 and 1640 cm^–1^ at the PCB82 exciton maximum and 1590 cm^–1^ at DBV exciton maximum. Further pump-probe study in a high magnetic field (25T)^45^ revealed that only the peak at 1580 cm^–1^ is shifted in the presence of an external magnetic field, while the 1640 cm^–1^ peak remains almost unperturbed, indicating significant mixing of the former with the electronic state. Similarly, in our transient IR spectra (Figure 2), we see contrasting behavior for the 1588 and 1619 cm^–1^ peaks. The 1588 cm^–1^ peak shows a sub-ps decay while the 1619 cm^–1^ peak doesn’t show any decay within the 100 ps experimental time window. This again shows that the 1588 cm^–1^ vibration gains DBV electronic character via vibronic mixing but the 1619 cm^–1^ peak is purely vibrational. This is primarily because the former is resonant to the DBV-PCB82 energy gap (1580 cm^–1^) while the latter is off-resonant.^11^ In addition, the C=NH^+^ group assigned to the 1588 cm^–1^ peak is spatially closer to DBV than the COOH group assigned to the 1619 cm^–1^ peak, facilitating the former’s participation in vibronic mixing.

## Conclusions

We take advantage of the high-resolution of 2DEV spectroscopy to resolve the exciton-specific vibrational structure and identify the characteristic ground and excited mid-IR signatures of each pigment in PC645. The protein structure provides insight into the pigment–protein interaction and enables assignment of each pigment-specific IR band, summarized in Figure 6. Due to the low signal-to-noise ratio and high photodegradation, it was not possible to collect the transient 2DEV spectra at more than a few selected waiting times. Therefore, we use transient IR spectra to obtain the excitation relaxation timescale, which agrees with previous visible-pump-probe studies.^10,35^ The combined transient IR and 2DEV spectral study provides knowledge about the marker mid-IR signatures of each exciton. This knowledge can potentially be used to track the energy flow between the exciton pairs of interest to enable insight into the influence of the vibronic interactions in the spatial flow of excitation energy within PC645. The infrared peaks specific to PCB82-C are useful to distinguish the dynamics within (<ps) and between (>ps) the two monomeric units. In particular, the C=N stretching strongly coupled with N–H bending mode in PCB82, resonant to DBV-PCB82 excitonic energy gap, is the potential candidate for the vibronic interaction leading to the sub-ps down-conversion of the excitation energy, necessary for high-efficiency transfer to chlorophyll-based complexes.

## Supporting Information Available

Detailed discussion on plausible pigment–protein interactions resulting in frequency shift of the mid-IR fingerprint signatures of each phycobilin pigment (PDF)

## Abbreviations

PC645: phycocyanin 645
IR: infrared
NOPA: noncollinear optical parametric amplifier
DBV: dihydrobiliverdin
MBV: mesobiliverdin
PCB: phycocyanobilins
2DEV: two-dimensional electronic vibrational
