# Supplementary material for: Infrared Signatures of Phycobilins within the Phycocyanin 645 Complex
Source: J Phys Chem B. 2023 May 16;127(20):4460–9. doi: 10.1021/acs.jpcb.3c01352 (PMC10226103; doi:10.1021/acs.jpcb.3c01352)
Supplement: Supplementary file 1 — jp3c01352_si_001.pdf [file jp3c01352_si_001.pdf]

Supporting Information

Infrared Signatures of Phycobilins within the Phycocyanin 645 Complex

Partha Pratim Roy[1,2,‡], Cristina Leonardo[1,2,‡], Kaydren Orcutt[1,2,‡], Catrina Oberg[3], Gregory D. Scholes[3], and Graham R. Fleming[1,2,4,]*

[1]Department of Chemistry, University of California, Berkeley, CA 94720, United States.

[2]Molecular Biophysics and Integrated Bioimaging Division, Lawrence Berkeley National Laboratory, Berkeley, CA 94720, United States.

[3]Department of Chemistry, Princeton University, Washington Road, Princeton, NJ 08540, United States.

[4]Kavli Energy Nanoscience Institute at Berkeley, Berkeley, CA 94720, United States.

Pigment-protein interaction

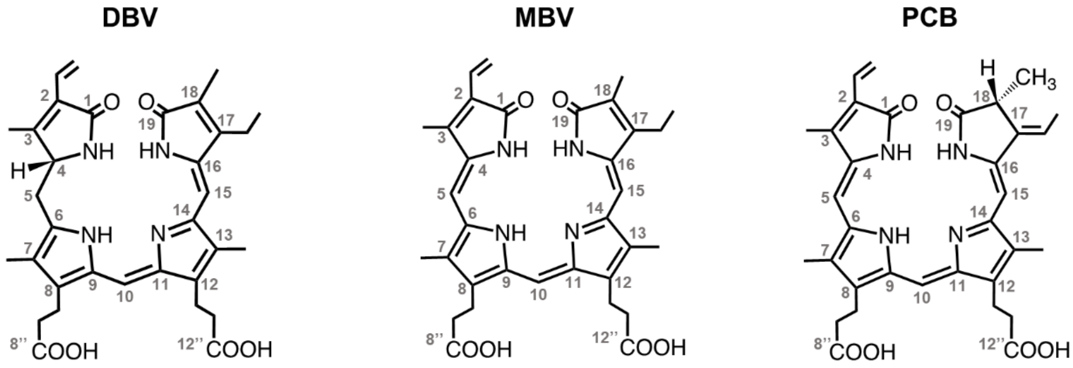

Figure S1. Chemical structures of the three main biliverdin pigments: dihydrobiliverdin (DBV), mesobiliverdin (MBV) and phycocyanobilins (PCB).

In this work, we identify the vibrational frequencies of C=N, C=C and C=O stretching modes of each pigment within PC645. The infrared signatures depend on the different electrostatic interactions between each pigment and the protein environment.[1] Here we list the hydrogen bonds using the RCSB Protein Data Bank structure (PDB ID: 4lms)[2] (Figure S2).

The 1C=O group of one unsaturated γ-lactams group of PCB82-C has a hydrogen bond with a cysteine residue (Figure S2c). The N-H bond of the same group has a hydrogen bond with n-methyl asparagine residue. Most carboxylic groups of PCB82-C and D form hydrogen bonds with arginine residues (Figure S2b-c). The 1C=O group in the lactam rings of both PCB158 pigments form hydrogen bonds with lysin residues, while both the 8’’COOH groups interact with arginine residues (Figure S2d-e). The carboxylic groups of both MBVs form hydrogen bonds with histidine or lysin and tyrosine residues (Figure S2f-g). The carboxylic group 8’’COOH of DBV-C forms a hydrogen bond with an arginine residue (Figure S2i).

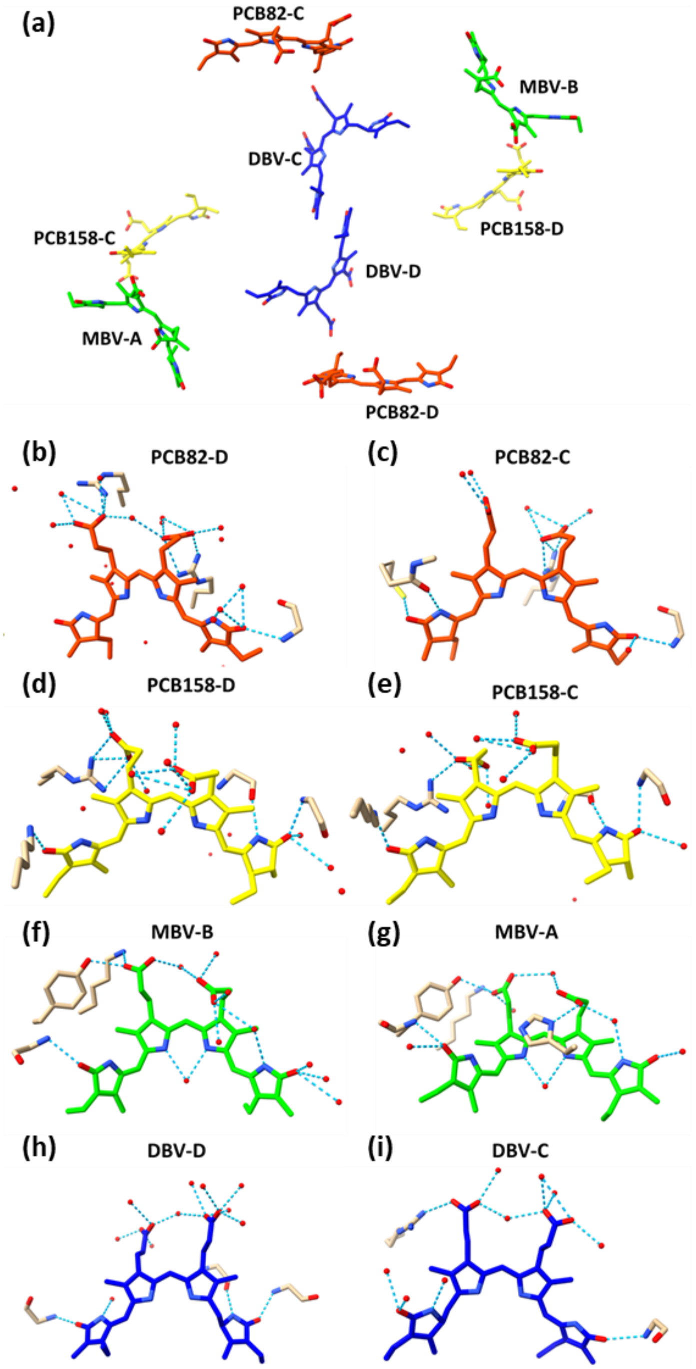

Figure S2. (a) Pigment arrangement in PC645 (PDB: 4lms).[2] Hydrogen bonds with water molecules and protein chain for (b) PCB82-D, (c) PCB82-C, (d) PCB158-D, (e) PCB158-C, (f) MBV-B, (g) MBV-A, (h) DBV-D and (i) DBV-C.

	1C=O	19C=O	8''COOH	12''COOH	1C=NH+	6C=NH+	14C=N	19C=N
**PCB82-D**	*ASP*		**ARG**	**ARG**				
**PCB82-C**	**CYS**	*ASP*		**ARG**	**MEN**			
**PCB158-D**	**LYS**	*GLY*	**ARG**			*PRO*		
**PCB158-C**	**LYS**	*GLY*	**ARG**			*PRO*		
**MBV-A**	*TYR*		**HIS**	**LYS/ TYR**				
**MBV-B**	*TYR*		**LYS/ TYR**					
**DBV-D**	*ILE*	*GLN*			*PRO*			
**DBV-C**		*ILE*	**ARG**					

Table S1 - The amino acid sidechains (bold) and main protein chains (italics) involved in pigment-protein hydrogen-bonds. Amino-acid residues: ASP – asparagine; ARG – arginine; CYS – cysteine; MEN – n-methyl asparagine; LYS – lysine; GLY – glycine; PRO – proline; TYR – tyrosine; HIS – histidine; ILE – isoleucine; GLN – glutamine. Numbers on carbon corresponds to Figure S1.

Noise floor and error bar of 2DEV experiments

Noise floor (Figure S3a) is calculated by integrating the 2DEV data outside the excitation spectral window, which gives average noise level of 0.097. Such a high signal-to-noise ratio was achieved by averaging a large number (16000) laser shots to collect a 2DEV spectrum for a selected waiting time. Figure S3b and S3c show the 2DEV spectral slices obtained from three independent measurements illustrate constituency of the 2DEV measurements. Slight change in amplitude from one measurement to other can be caused by the error in concentration during sample preparation as well as excitation laser power fluctuations during measurements.

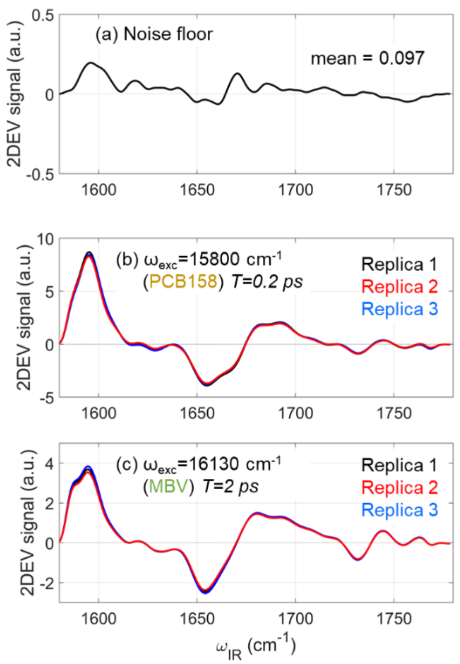

Figure S3: (a) Average noise floor for 2DEV measurement. 2DEV spectral slices at (b) ω_{exc}=15800 cm^{-1}, T=0.2 ps and (c) ω_{exc}=16130 cm^{-1}, T=2 ps for three independent measurements are shown by black, red and blue colors.

References

(1) Toa, Z. S. D.; Dean, J. C.; Scholes, G. D. Revealing Structural Involvement of Chromophores in Algal Light Harvesting Complexes Using Symmetry-Adapted Perturbation Theory. *J. Photochem. Photobiol. B Biol.* **2019**, *190*, 110–117. https://doi.org/10.1016/j.jphotobiol.2018.11.007

(2) Harrop, S. J.; Wilk, K. E.; Dinshaw, R.; Collini, E.; Mirkovic, T.; Teng, C. Y.; Oblinsky, D. G.; Green, B. R.; Hoef-Emden, K.; Hiller, R. G.; et al. Single-Residue Insertion Switches the Quaternary Structure and Exciton States of Cryptophyte Light-Harvesting Proteins. *Proc. Natl. Acad. Sci. U. S. A.* **2014**, *111* (26). https://doi.org/10.1073/pnas.1402538111.
